# Genome Sequence of Micromonospora aurantiaca Strain G9, a Member of a Bacterial Consortium Capable of Polyethylene Degradation

**DOI:** 10.1128/mra.01148-21

**Published:** 2022-04-07

**Authors:** Beate Schneider, Friedhelm Pfeiffer, Mike Dyall-Smith, Hans-Jörg Kunte

**Affiliations:** a Bundesanstalt für Materialforschung und -Prüfung, Division of Biodeterioration and Reference Organisms, Berlin, Germany; b Computational Biology Group, Max Planck Institute of Biochemistry, Martinsried, Germany; c Veterinary Biosciences, Faculty of Veterinary and Agricultural Sciences, University of Melbourne, Parkville, Australia; University of Maryland School of Medicine

## Abstract

Nine different bacterial isolates were recovered from landfills. Each isolate was obtained in pure culture. As a consortium, the bacteria degrade polyethylene. The complete genome sequence of strain G9 was determined by PacBio sequencing. Using the TYGS server for taxonomic classification, strain G9 was assigned to the species Micromonospora aurantiaca.

## ANNOUNCEMENT

As part of a consortium of nine bacterial isolates, strain Micromonospora aurantiaca G9 contributes to the degradation of polyethylene film and was isolated from a former landfill site for plastics (Niemegk-Neuendorf, Germany 52°04′59″N, 12°41′59″E) by applying a modified iChip procedure (1). A bacterial suspension obtained from the landfill was diluted in soft agar (0.6%) and filled into the chambers of an iChip. The iChip was sealed with a polycarbonate membrane, deposited in soil, and incubated for 4 weeks. The emerging microcolony of strain G9 was isolated, and cells from a single colony were inoculated into liquid N-Z-amine medium (medium 554; DSMZ, Germany) without CaCO_3_. Cells were grown aerobically at 28°C for 24 h and then supplemented with 250 mL concentrated medium 554 (2×) to increase the cell mass. After growing for another 24 h, cells were centrifuged and washed twice with phosphate-buffered saline (PBS). Cell pellets were frozen in liquid nitrogen and kept at −80°C.

The cell pellets were sent to Genewiz/Azenta (Leipzig, Germany) for DNA extraction using the Genomic-tip 100/G (Qiagen, Hilden, Germany) following the manufacturer’s protocol. DNA was sheared to ∼10 kb using g-TUBEs without size selection. After quality (NanoDrop and pulsed-field gel electrophoresis) and quantity (Qubit 2.0) assessment, the sheared DNA was used for sequencing library preparation using the SMRTbell library preparation kit v2.0 (Pacific Biosciences [PacBio], Menlo Park, CA, USA) according to the manufacturer’s instructions. The library was sequenced on a PacBio Sequel sequencer (2) using one PacBio Sequel single-molecule real-time (SMRT) Cell. The number of reads was 105,886, from which 772,196 subreads were obtained (average read length, 3,960 bp; read *N*_50_, 5,692 bp; total size, 3,057 Mbp). Within the SMRT Link Suite v5.0.0.6792, a supervised assembly was performed at Genewiz/Azenta using a proprietary method; this included checks for quality and coverage, subread coverage reduction by random downsampling to 200-fold, and automated genome assembly (HGAP v4, which calls the assembler Canu [3]). In Canu, the coverage cutoff was set to 40-fold. The assembly was evaluated, overlapping contigs were merged, and potential assembly artifacts were dropped. If appropriate, contigs were circularized using Circlator v1.5.5 (4). Contigs were polished with raw subreads using Arrow. This assembly resulted in 21 contigs, with a total length of 7,261,531 bp.

The assembly was inspected manually using Geneious v10.2 (5). The largest contig, obtained with 395-fold average coverage, represents the complete and circular chromosome of 7,149,080 bp, with a G+C content of 72.8%. The remaining smaller contigs were considered irrelevant (e.g., misassemblies with minimal coverage), confirming that the organism has a single chromosome and no plasmids. The termini of the assembled sequence were validated against GenBank accession number CP002162 (Micromonospora aurantiaca ATCC 27029). The start base was shifted manually (Emacs editor in Linux) to match that of GenBank accession number CP002162 and to be upstream of the *dnaA* gene.

The TYGS (6) assigned strain G9 to the species Micromonospora aurantiaca, with a digital DNA-DNA hybridization (dDDH) (d_4_) value of 89.5% (95% confidence interval, 87.1 to 91.5% [7]). The G+C content difference of 0.03% is minimal compared to type strain Micromonospora aurantiaca ATCC 27029 and supports the assignment. The genome was submitted to GenBank and annotated using PGAP v5.3 (8).

### Data availability.

The annotated genome has been deposited in GenBank under the BioProject accession number PRJNA768672 and the BioSample accession number SAMN22060555. The nucleotide sequence accession number is CP084582. The Sequence Read Archive (SRA) accession number is SRX12530604.
